# Editorial: The development and utilization of novel antibiotic alternatives

**DOI:** 10.3389/fmicb.2022.1008850

**Published:** 2022-08-31

**Authors:** Wang Jiajun, Li Wenyu, Kianoush KHosravi-Darani, In Ho Kim

**Affiliations:** ^1^Department of Animal Nutrition, Institute of Animal Nutrition, Northeast Agricultural University, Harbin, China; ^2^National Nutrition and Food Technology Research Institute, Faculty of Nutrition Sciences and Food Technology, Shahid Beheshti University of Medical Sciences, Tehran, Iran; ^3^Department of Animal Resource & Science, Dankook University, Yongin, South Korea

**Keywords:** antibiotic alternatives, antimicrobial peptides, endolysin, probiotics, phytochemicals

The discovery of antibiotics is one of the greatest milestones in the history of human medical, meanwhile, that have promoted the rapid advancement in animal production. Statistics predict that up to 200,235 tons of antibiotics will be used in food animals to promote the growth of animals and to prevent bacterial infections in 2030, but most of them are not absorbed by animals and are excreted *via* feces and urine, which can directly contaminate and harm the surrounding environment (Xu et al., 2022; Li et al.). Even worse, the heavy misuse of antibiotics undoubtedly also contributes to the emergence of multidrug-resistant (MDR) bacteria, and results in a potential global health crisis. To tackle the resistance problems, countries have gradually restricted or banned the use of antibiotics in feed in recent years (Innes et al., 2020). Under this background, the development of antibiotic alternatives is a top priority to ensure the healthy development of animal production. In this Research Topic, a total of 14 excellent articles presenting different proposals of antibiotic alternatives are included.

## Antimicrobial peptides

Antimicrobial peptides, as small molecule peptides produced by the host to resist the infections of external pathogens, are one of the most promising antibiotic alternatives based on their membrane disruption mechanisms and immunomodulatory effects (Wang et al., 2019a). Despite their potent activity as antimicrobials, AMPs still face significant challenges toward therapeutic applications, such as overall high toxicity and relatively poor metabolic stability, whilst the latter directly determines that most antimicrobial peptides are mainly administrated topically (Wang et al., 2019b; Nepal et al.).

Currently, numerous chemical modifications have been proposed to improve the metabolic stability of AMPs, among which, peptidomimetic approaches can reduce proteolytic susceptibility by removing the peptide-like characters of AMPs thereby resulting in the inability of proteases to recognize and degrade the resulting mimic molecules (Domalaon et al., 2016). Ramirez et al. designed a non-hemolytic peptidomimetic dUSTBβP 3, comprised of three β^3^-homoarginine residues and two fatty acyl tails eight carbons long. In comparison to the α-amino acid-based counterpart, the compound 3 showed excellent *in vitro* plasma stability with an extrapolated half-life of 10.43 ± 3.70 h. Notably, dUSTBβP 3 could potentiate the activity of novobiocin and rifampicin against wild-type and multiple drug resistance (MDR) Gram-negative bacteria and reduce their minimum inhibitory concentrations below the interpretative susceptibility breakpoints.

In recent years, dietary modulation of endogenous AMPs synthesis has been continuously studied as a new antibiotic alternative approach to avoid the application problems of exogenous AMPs. Yang et al. investigated a potential synergy between sugars and butyrate in inducing HDP gene expression in chickens, and found that sugars differentially regulated HDP expression in both gene- and sugar-specific manners in chicken HD11 macrophage cells. Importantly, these sugars could synergize with butyrate to further enhance chicken HDP and barrier function gene expression to effectively control and prevent infectious diseases.

## Endolysin

Bacteriophages, which are the natural predators of bacteria, have been reactivated for combating the continuing development of antibiotic resistance. In fact, bacteriophages-therapy has intrinsic disadvantages, one of which is that they cannot be administered intravenously since they are destroyed by the immune system (Bassetti et al., 2017). Thus, the endolysin itself has been proposed to instead of living phages as antibacterial agents. Vasina et al. provided a comprehensive study on the biological function of four Gram-negative bacteria-targeting endolysins LysAm24, LysAp22, LysECD7, and LysSi3. These endolysins possessed a wide antibacterial spectrum against planktonic bacteria and bacterial biofilms, and were effective in wound and burn skin infection animal models. In terms of safety, these endolysins did not contribute to the development of short-term resistance, with no cytotoxicity and no significantly effects on the normal intestinal microflora *in vivo*.

## Probiotics

Probiotics, also known as live bacteria preparations or micro-ecological preparations, are increasingly popular as feed additives and alternatives to traditional antibiotics. Among numerous probiotics, the spore-forming *Bacillus* species are considered to be more suitable for application in the feed industry, in comparison to non-spore-forming probiotics (Bernardeau et al., 2017), but it is still unknown whether the probiotic functions of the *Bacillus* depend on the germination of spores *in vivo*. Addressing this issue, Lu et al. detected the germination response of 14 *Bacillus* spores in relation to different germinating agents, and revealed that the germination response was strain-specific and germinant-related, and demonstrated that the germination of spores initiated by L-alanine could result in an increased probiotic effects.

Metabolites are also one of underlying mechanisms by which probiotics exert antibacterial and immunomodulatory activities. Thus, Møller et al. utilized metabolomics analysis to explore the mechanisms by which *Lactobacillus* spp. Lb21 protected *C. elegans* against MRSA, and successfully identified a set of metabolites that potentially could lead to strategies for protection against MRSA.

## Phytochemicals

Phytochemicals have been used for many years to treat various ailments (Li et al., 2021), which possess many unique advantages compared with the above several antibiotic alternatives, such as exceptionally rich sources, diverse chemical structures, and multiple biological functions (Porras et al., 2021). Therefore, phytochemicals are gaining significant attention as substitutes for antibiotics.

Plant polyphenolic compounds, as a large family of phytochemicals widely distributed in the plant kingdom, have won the favor of many researchers because of their variety of biological functions including anti-carcinogenic, anti-inflammatory, and anti-oxidant properties. Moreover, some phenolic compounds have been proven to be effective in inhibiting various pathogenic bacteria, such as *E. coli* (Bancirova, 2010), halitosis-related bacteria (Liu et al.) and so on. Currently, the exact antibacterial mechanisms of polyphenolic compounds are still unclear, but some studies have demonstrated that polyphenolic compounds can cause disruption in bacteria cell membranes, for instance, Zhang et al. confirmed that the compound phenolic acid (protocatechuic acid, hydrocinnamic acid, and chlorogenic acid) could cause bacterial flagellar abscission, cell membrane structure damage, and finally lead to the leakage of intracellular macromolecules, and bacterial death.

In addition to polyphenolic compounds, natural plant essential oils (EOs) also have been well-known as potential microbicides. However, bactericidal efficacies of EOs are controversial, mainly because the amount of active ingredients in individual EOs varies with sources and extraction process. Lu et al. comprehensively analyzed the main components of oregano essential oil (OEO) and found carvacrol and thymol are the major ingredients responsible for its bactericidal activity. Moreover, carvacrol/thymol could synergize with 405 nm blue light (BL) to kill multidrug-resistant *Pseudomonas aeruginosa*, greatly improving their antibacterial potential. These findings offered a unique opportunity to standardize OEO products in the basis of the amount of carvacrol and thymol.

Due to their excellent properties as antibiotic alternatives, various phytochemicals have been used in animal production as feed additive to improve animal health and growth performance (Li et al., 2021; Ayalew et al., 2022), and some have been successfully commercialized. In this topic, Yin et al. demonstrated that dietary *Lycium barbarum* polysaccharides (LBPs) supplementation could improve growth performance, antioxidant capacity and immunity, and reduced diarrhea incidence in weaned piglets. Koorakula et al. systematically studied the impacts of a commercial phytogenic feed additives (PFAs) product on the gut microbiome and resistome of broiler chickens. The results revealed that PFA treatments increased the abundance of *Firmicutes* such as *Lactobacillus*, reduced the abundance of *Escherichia*, whilst resulted in a decrease in abundance of antibiotic resistance gene. Taken together, phytochemicals have very broad application prospects as feed additives to replace antibiotics in animal production.

## Dihydropyrimidinones derivatives

DHPMs are nitrogen-containing heterocyclic substances with excellent pharmacological activities such as antitumor, antiviral, anti-inflammatory and so on. On this basis, Jara et al. further developed three DHPM derivatives with potent antibacterial activity against MDR bacteria and no significant cytotoxicity. It seems that the development of derivatives based on existing antibacterial substances is also an effective mean to expand the library of antibiotic alternatives.

In conclusion, in face of the increasing number of MDR bacteria, the development of new antibiotic alternatives, both for animal production and human health, is imperative. We hope that this Research Topic will be useful for further research in this field.

## Author contributions

All authors listed have made a substantial, direct, and intellectual contribution to the work and approved it for publication.

## Funding

This work was supported by the National Natural Science Foundation of China (32002215) and the Natural Science Foundation of Heilongjiang Province (YQ2020C009).

## Conflict of interest

The authors declare that the research was conducted in the absence of any commercial or financial relationships that could be construed as a potential conflict of interest.

## Publisher's note

All claims expressed in this article are solely those of the authors and do not necessarily represent those of their affiliated organizations, or those of the publisher, the editors and the reviewers. Any product that may be evaluated in this article, or claim that may be made by its manufacturer, is not guaranteed or endorsed by the publisher.
